# Utah Latina/o/x suicide decedents less likely to die by firearm, even in rural areas: examining population-wide data from the Utah Office of the Medical Examiner

**DOI:** 10.3389/fpubh.2024.1358043

**Published:** 2024-04-10

**Authors:** Douglas Tharp, Evan V. Goldstein, Richard M. Medina, Simon C. Brewer, Amanda V. Bakian, Hilary Coon

**Affiliations:** ^1^Department of Geography, University of Utah, Salt Lake City, UT, United States; ^2^Department of Population Health Sciences, Spencer Fox Eccles School of Medicine, University of Utah, Salt Lake City, UT, United States; ^3^Department of Psychiatry, Huntsman Mental Health Institute, University of Utah, Salt Lake City, UT, United States; ^4^Department of Family and Preventive Medicine, Spencer Fox Eccles School of Medicine, University of Utah, Salt Lake City, UT, United States

**Keywords:** suicide, firearms, self-injurious behavior, rural health, health inequities, Hispanic/Latino

## Abstract

**Introduction:**

Suicide death remains a significantly rarer event among Latina/o/x populations compared to non-Latina/o/x populations. However, the reasons why Latina/o/x communities experience relatively lower suicide rates are not fully understood. Critical gaps exist in the examination of Latina/o/x suicide death, especially in rural settings, where suicide death by firearm is historically more common within non-Latina/o/x populations.

**Method:**

We tested whether the prevalence of Latina/o/x firearm suicide was meaningfully different in urban and rural environments and from non-Latino/a/x decedents when controlling for age, sex, and a social deprivation metric, the Area Deprivation Index. Suicide death data used in this analysis encompasses 2,989 suicide decedents ascertained in Utah from 2016 to 2019. This included death certificate data from the Utah Office of the Medical Examiner on all Utah suicide deaths linked to information by staff at the Utah Population Database.

**Results:**

Compared to non-Latina/o/x suicide decedents, Latina/o/x suicide decedents had 34.7% lower adjusted odds of dying by firearm. Additionally, among the firearm suicide decedents living only in rural counties, Latina/o/x decedents had 40.5% lower adjusted odds of dying by firearm compared to non-Latina/o/x suicide decedents.

**Discussion:**

The likelihood of firearm suicide death in Utah differed by ethnicity, even in rural populations. Our findings may suggest underlying factors contributing to lower firearm suicide rates within Latina/o/x populations, e.g., aversion to firearms or less access to firearms, especially in rural areas, though additional research on these phenomena is needed.

## Introduction

Suicide death has become more common in Latina/o/x^1^ communities over the past decade, with age-adjusted Latina/o/x suicide rates in the United States increasing by 38.4% from 2013 to 2021 (1). However, suicide death remains a significantly rarer event among Latina/o/x individuals compared to non-Latina/o/x individuals. Although rates of suicidality and suicide risks among Latina/o/x individuals can vary across nationalities and between U.S.- and foreign-born populations (2–4), age-adjusted suicide rates among Latina/o/x individuals overall were 51.6% that of non-Latina/o/x individuals in 2021 (7.89 and 15.28/100,000 persons, respectively) (1). Silva and Van Orden (2) have summarized that specific social and cultural factors may increase the likelihood of suicidality and psychological distress within different Latina/o/x populations, such as acculturation challenges (5) and discrimination (6). Previous studies using indicators of poverty have also suggested that Latina/o/x populations may experience higher rates of suicide death and suicidality when they are economically worse off (7, 8). Nevertheless, the reasons why Latina/o/x populations have historically experienced relatively lower suicide rates than non-Latina/o/x populations are not fully understood (2).

Critically, little is known about the epidemiology of firearm suicide death within Latina/o/x populations (9). In general, access to firearms – the most common and lethal method of suicide death (10, 11) – exacerbates suicide risk (12, 13). Many people who attempt suicide ultimately survive (14); however, survival is typically less likely for suicide attempts involving firearms, given the 80–90% case-fatality rate of firearm attempts (15, 16). Firearm suicide rates among Latina/o/x individuals increased by 57.9% from 2013 to 2021 nationally (1). In 2021, firearms accounted for a majority of suicide deaths in the U.S. overall (1) but only 41.5% of suicide deaths among Latina/o/x individuals (17). The next most common mechanisms of suicide death among Latina/o/x individuals nationally were suffocation and drug-related poisoning, accounting for 39.6 and 7.3% of suicide deaths among Latina/o/x individuals, respectively, in 2021 (17). The high lethality of firearm suicide attempts and recent evidence indicating an increase in firearm ownership among Latina/o/x individuals (18, 19) therefore pose significant but ambiguous public health and prevention challenges.

Further exacerbating these challenges is the lack of knowledge about firearm suicide death among Latina/o/x individuals who live in rural (or non-metropolitan) areas. Suicide rates tend to be higher in rural areas than urban areas (20), notably among non-Latina/o/x individuals (1). Historically, higher rates of suicide death in rural areas are thought to be the product of multiple contextual factors, such as interpersonal isolation and economic distress (21, 22). More recent studies suggest that these rural–urban differences are driven by greater firearm use in rural areas (i.e., more firearm-related suicide deaths) (23). Specifically, Nestadt et al. underscored the important role of firearm use in rural suicide mortality (23). The authors found that, although there was a higher suicide rate in rural Maryland counties than urban counties, the difference in the suicide rate was limited to firearm suicide deaths and did not exist when only evaluating non-firearm suicide deaths. Other recent studies examining large, national samples of firearm suicide decedents have indicated that Latina/o/x and non-Latina/o/x firearm suicide decedents typically differ demographically (e.g., younger) (9). However, despite Latina/o/x individuals currently representing the largest share of the rural minority population in the U.S. (24), previous studies have not specifically investigated whether or why firearm suicide rates differ between Latina/o/x and non-Latina/o/x individuals in rural areas. Understanding firearm suicide death in rural Latina/o/x populations may help refine the delivery of specific interventions, such as lethal means assessment and counseling, better informing when, where, and for whom the interventions should be delivered.

To address these gaps in the literature, we examined detailed data from suicide decedents in Utah from 2016 through 2019. In Utah, about 15.1% of the population identifies as Latina/o/x (25), and the rate of household firearm ownership in Utah has been above the national average for the past four decades (26). Investigating this population and its firearm attitudes and behaviors [e.g., potential firearm aversion, as revealed in previous survey research (27)] is key to understanding changing suicide rates. Specifically, we estimated the relationship between Latina/o/x ethnicity and suicide death involving firearms. We also tested whether the prevalence of firearm suicide deaths differed among Latina/o/x decedents living in rural versus urban counties.

## Methods

This study used data from the Utah Suicide Mortality Risk Study (USMRS), which has been described in detail elsewhere (28, 29). Briefly, this study is possible through a long-term collaboration with the Utah State Office of the Medical Examiner (OME). Suicide status was determined by the OME after a detailed investigation of the scene of death, interviews with next-of-kin, and circumstances of death. Official death certificate data from the OME on all Utah suicide deaths was securely linked to information by staff within the Utah Population Database (UPDB), e.g., information from various vital records and additional sources. With data on >12 million individuals, the UPDB at Huntsman Cancer Institute at the University of Utah is one of the world’s richest sources of in-depth information supporting demography and public health research (30). The UPDB provides access to information that can only be used for biomedical and health-related research; the privacy of individuals represented in its records and confidentiality of the data are strictly protected. UPDB staff at the University of Utah conducted the secure linkage of information within the UPDB for the suicide decedents included in this study. The death data used in this analysis encompasses 2,989 suicide decedents ascertained population-wide in Utah from 2016 to 2019, the last pre-pandemic years. The Institutional Review Boards at the University of Utah, Intermountain Healthcare, and the Utah Department of Health approved the parent study.

### Variables

Our dependent variable was a binary measure of firearm suicide death (yes, no), determined by the OME after a detailed investigation of the scene, interviews with next-of-kin, and circumstances of death. Our independent variables were binary indicators of decedent ethnicity (Latina/o/x, non-Latina/o/x) and county rurality (rural, urban), based on the county of residence for each decedent. Decedent ethnicity was recorded by the OME after being identified by the death investigator and finally determined by the decedent’s family through a conversation with the next-of-kin and/or funeral director. Latina/o/x decedents included individuals who identified as having Mexican, Puerto Rican, Cuban, Central or South American, or other Spanish/Portuguese culture or origin, e.g., regardless of race. We defined urban as residence in one of seven counties (Salt Lake, Utah, Davis, Weber, Tooele, Juab, Morgan) classified as metropolitan with code 1 or 2 in the 2023 Rural–Urban Continuum Codes system maintained by the U.S. Department of Agriculture (31). The Rural–Urban Continuum Codes system distinguishes metropolitan (urban) counties by the population size of their metro area, and nonmetropolitan (rural) counties by their degree of urbanization and adjacency to a metro area. All residences not in these counties were classified as rural.

Additional demographic covariates included sex and age. Age at death, the only non-binary variable, was logged to improve model fit. We also logged and included a measure of Area Deprivation Index (ADI) in our multivariable analyses (32). Our measure of ADI was created by the Health Resources & Services Administration over three decades ago and has since been adapted and validated to the neighborhood level by researchers at the University of Wisconsin-Madison (33). This powerful composite measure allows for ranking area-level residence by socioeconomic disadvantage and includes factors across multiple theoretical domains, including income, education, employment, and housing quality.

### Statistical analysis

We first estimated a generalized linear model (GLM) with a binomial distribution and the logit link function to test the association between Latina/o/x ethnicity and death by firearm. We then estimated a second GLM to test this relationship for only rural decedents. We present the regression model coefficients as odds ratios (i.e., exponentiated log-odds) for ease of interpretation. We also tested differences in the prevalence of rural and urban Latina/o/x firearm suicide decedents using a chi-square test with Yates’ continuity correction. All analyses were conducted using R (Version 4.0.5, RStudio Version 1.3.1093).

## Results

Organized by the rate of suicide, firearms were the second most common method of suicide death among Latina/o/x decedents (3.7/100,000 persons; Table 1) and the most common method of suicide death among non-Latina/o/x decedents (11.8/100,000 persons) in Utah over the study period. Compared to non-Latina/o/x suicide decedents, Latina/o/x suicide decedents had 34.7% lower odds of dying by firearm after adjusting for the covariates described above (Model 1; aOR = 0.653; 95% CI 0.486–0.875; Table 2). On average, decedents living in urban counties had 31.5% lower odds of dying by firearm suicide compared decedents living in rural counties (aOR = 0.685; 95% CI 0.580–0.808). Male decedents were more than three times as likely as female decedents to die by firearm suicide (aOR = 3.229; 95% CI 2.644–3.913) after adjusting for the other variables in the model. Increasing age was also independently associated with a greater likelihood of dying by firearm suicide (aOR = 1.481; 95% CI 1.242–1.768). Greater area deprivation was not associated with a greater likelihood of dying by firearm suicide.

**Table 1:** Suicide deaths per 100,000 persons by method of suicide death and ethnicity in Utah: 2016–2019.

	Latina/o/x			Non-Latina/o/x		
	Per 100,000 persons	*N*	%	Per 100,000 persons	*N*	%
Firearm	3.7	73	39%	11.8	1,191	53%
Asphyxiation	4.3	84	44%	6.5	667	30%
Overdose	0.9	18	10%	2.8	282	13%
Violent trauma	0.6	12	6%	0.9	86	4%
Other	0.1	2	1%	0.2	19	1%

Authors’ analysis of data from the OME.

**Table 2:** Odds ratios from multivariable generalized linear models estimating the relationship between suicide death by firearm and decedent ethnicity in Utah: 2016–2019.

	Model 1: Full model	Model 2: Rural decedents only
	aOR (95% CI)	aOR (95% CI)
*Ethnicity (Ref: Non-Latina/o/x)*		
Latina/o/x	0.653	0.596
	(0.486–0.875)	(0.339–0.930)
*County Rurality (Ref: Rural)*		
Urban	0.685	
	(0.580–0.808)	
*Sex (Ref: Female)*		
Male	3.229	3.34
	(2.644–3.913)	(2.463–4.569)
Age at death (logged)	1.481	1.513
	(1.242–1.768)	(1.412–2.009)
Area deprivation index (logged)	0.951	1.039
	(0.822–1.100)	(0.821–1.311)
(Intercept)	0.164	0.109
	(0.070–0.385)	(0.028–0.419)

Authors’ analysis of data from the OME. 95% confidence intervals are shown in parentheses. aOR is adjusted odds ratio (i.e., exponentiated log-odds). Model 1 included all suicide decedents in our analytic sample (*n* = 2,676). Model 2 included all suicide decedents living in rural counties (*n* = 1,005). Taking the log of the odds ratios for the age variable will provide the estimates in log-odds form. An increase in age is associated with a greater propensity to die by firearm (i.e., aOR > 1).

### Likelihood of firearm suicide death among Latina/o/x individuals in rural Utah

The prevalence of firearm suicide death in rural and urban counties differed by ethnicity (*p* < 0.001; Table 3). Consistent with previous studies demonstrating higher firearm suicide rates in rural counties (23), a majority (58.3%) of non-Latina/o/x suicide decedents living in rural counties died by firearm, compared to 49.9% of non-Latina/o/x suicide decedents living in urban counties. In contrast, however, less than half (45.2%) of Latina/o/x suicide decedents living in rural counties died by firearm. After adjusting for our other covariates, among the firearm suicide decedents living only in rural counties, Latina/o/x decedents had 40.5% lower odds of dying by firearm compared to non-Latina/o/x suicide decedents (Table 2; Model 2; aOR = 0.596; 95% CI 0.339–0.930).

**Table 3:** Percent of suicide deaths involving firearms by ethnicity and county rurality in Utah: 2016–2019.

	Latina/o/x	Non-Latina/o/x	*p*-value
County rurality			<0.001
Rural	45.2%	58.3%	
Urban	38.2%	49.9%	

Authors’ analysis of data from the OME.

## Discussion

Suicide death remains rarer – with reported suicide rates being nearly 50% lower – among Latina/o/x individuals compared to non-Latina/o/x individuals in Utah (1). Taken together, two key findings from this study may offer insight into why Latina/o/x communities do not experience the same burden of suicide death as the non-Latina/o/x white population in Utah. First, Latina/o/x suicide decedents had about 34.7%% lower adjusted odds of dying by firearm than non-Latina/o/x decedents in our study. This finding stands in contrast with the fact that firearm suicide rates have risen considerably (34.6%) overall in Utah since 2000 and that firearms account for more than half of all suicide deaths in Utah (1). Second, previous studies have demonstrated that firearm-related deaths account for higher suicide rates in rural areas compared to urban areas (23). When including both Latina/o/x and non-Latina/o/x suicide decedents, the findings from our multivariable analysis (Model 1) were consistent with those studies: Urban suicide decedents were significantly less likely to die by firearm than rural suicide decedents overall. However, we also found that firearms accounted for less than half of Latina/o/x suicide deaths in rural areas – and that Latina/o/x suicide decedents were significantly less likely to die by firearm than non-Latina/o/x suicide decedents in rural Utah after adjusting for other covariates in a multivariable analysis. For these reasons, our findings improve what is currently known about the epidemiology of firearm suicide death within Latina/o/x populations and suggest that there are underlying factors contributing to lower firearm suicide rates within Latina/o/x populations, especially in rural areas.

According to theories of suicidal behavior aligned with the *Ideation-to-Action* framework (34), the capability for suicide is influenced by contributors that facilitate an individual to attempt suicide, including *practical contributors* such as those that increase knowledge of and access to lethal means. Our findings may possibly suggest that Latina/o/x suicide decedents have an aversion to firearms or less access to firearms, especially in rural areas. Recent research has demonstrated that Latina/o/x individuals are less likely to be firearm owners than non-Latina/o/x individuals (18). Moreover, a recent public opinion poll conducted by Pew Research found that 73% of respondents identifying as “Latino” said it was more important to control firearms than protect the right to own firearms, compared to 52% of U.S. adults overall (27), and other public opinion polls have demonstrated similar findings (35). These results may indicate cultural aversions to firearms or even safer firearm storage practices shared by Latina/o/x community members. Equally important to consider, access to firearms within the Latina/o/x community may be minimized as a function of documentation or other social issues (e.g., comfort in purchasing from available firearm stores, refusal of sale, language barriers). Additional studies investigating potential protective factors related to firearm aversion and access will be critical for understanding firearm suicide death in Latina/o/x populations in Utah and beyond, especially as firearm suicide rates continue to rise among Latina/o/x individuals nationally (1), and given post-COVID-19-pandemic evidence indicating an increase in firearm ownership among Latina/o/x individuals (18, 19).

Another protective factor contributing to lower suicide rates within Latina/o/x populations may be Latina/o/x individuals’ ethnic identity and sense of belonging to a specific cultural group (2). Even in the presence of firearms or other lethal means, the *Interpersonal Theory of Suicide* posits that thwarted belongingness, where an individual perceives a disconnection in their closeness with others or a lack of reciprocity in caring, is a key antecedent to suicide (36). It may be that there is higher social cohesion or lower social isolation among Latina/o/x individuals in rural Utah, especially relative to urban-dwelling Latina/o/x Utahns (e.g., the urban counties along Utah’s Wasatch Front). Rural areas are traditionally thought to experience higher rates of suicide death due to the combination of multiple contextual factors, such as social isolation and economic distress (21, 22). Notably, though, the inclusion of ADI – an index which takes multiple factors from multiple theoretical domains into account, including income, education, employment, and housing quality – in our multivariable analyses did not explain significant variation in the likelihood of dying by firearm suicide. More research is therefore needed to investigate the demographic composition of rural Latina/o/x Utahns, the strength of their social networks, and the factors contributing to their firearm suicide rates being consistent with urban Latina/o/x Utahns. An additional factor to consider is that, while rural vs. urban populations are inherently different in certain ways, the differences may be more pronounced within Latina/o/x communities where rural activities are largely agrarian, some being guest workers. Such studies will have high population health importance, especially in states like Utah, where the Latina/o/x population is projected to triple in size by 2065 (37). From a public health perspective, the growing Latina/o/x population may depress suicide rates overall as their share of the weighted average increases. This should not divert our attention to further investigating possible reasons for why Latina/o/x populations experience relatively lower suicide rates, e.g., cultural antipathy to firearm possession or safer firearm storage practices.

### Limitations

This study had limitations. First, we could not account for firearm availability or attitudes, social factors, or health-related factors in our analyses. It is plausible that these or other unmeasured factors related to the well-known *Latina/o/x (Hispanic) paradox* may explain why Latina/o/x persons experience lower firearm and non-firearm suicide rates, in addition to living longer and experiencing lower annual mortality rates overall (38). An epidemiological finding, this paradox refers to the fact that Latina/o/x people generally have similar or better health outcomes than non-Latina/o/x despite often experiencing lower socio-economic status and education. Notably, cultural factors may increase the likelihood of suicidality and psychological distress within different Latina/o/x populations, such as acculturation challenges (5) and discrimination (6). We also only examined data from Utah, so our findings may not be generalizable to other states. Notably, the distribution of firearm suicide deaths among Latina/o/x individuals in rural and urban areas may differ in other states. For example, in 2020, Latina/o/x suicide deaths involving firearms were slightly more common in non-metropolitan counties (41.2%) than in metropolitan counties (38.9%) across the U.S., though Latina/o/x suicide deaths involving firearms occurred at similar proportions in non-metropolitan (51.2%) and metropolitan (50.0%) counties in the Mountain Census Division states alone (17). Additional studies, especially those comparing to living individuals, may yield insights into suicide risk factors or prevention pathways for Latina/o/x individuals in rural communities.

## Conclusion

Even though firearm use is believed to drive higher suicide rates in rural counties overall (23), we found that Latina/o/x suicide decedents were significantly less likely to die by firearm than non-Latina/o/x suicide decedents in rural Utah when adjusting for other covariates in a multivariable analysis. These findings likely contribute to explaining why Latina/o/x communities experience relatively lower suicide rates. Many people who attempt suicide ultimately survive (14); however, survival is typically less likely for those who use firearms, given the firearm method’s 80–90% case-fatality rate (15). Additional research on firearm suicide death in rural Latina/o/x populations may eventually help improve the delivery of specific interventions, such as lethal means assessment and counseling, along with better informing when, where, and for whom the interventions should be delivered.

## Data availability statement

The data analyzed in this study is subject to the following licenses/restrictions: the datasets presented in this article are not readily available due to privacy restrictions and the inclusion of vulnerable populations. The original contributions presented in the study are included in the article material, and further inquiries can be directed to the corresponding author. Requests to access these datasets should be directed to the corresponding author.

## Ethics statement

The studies involving humans were approved by the University of Utah, Intermountain Healthcare, and the Utah Department of Health. The studies were conducted in accordance with the local legislation and institutional requirements. The Ethics Committee/Institutional Review board waived the requirement of written informed consent for participation from the participants or the participants’ legal guardians/next of kin because deceased individuals included in the study could not be contacted.

## Author contributions

DT: Conceptualization, Data curation, Formal analysis, Methodology, Visualization, Writing – original draft. EG: Conceptualization, Methodology, Supervision, Writing – original draft, Writing – review & editing. RM: Conceptualization, Supervision, Writing – review & editing. SB: Methodology, Supervision, Writing – review & editing. AB: Methodology, Supervision, Writing – review & editing. HC: Project administration, Supervision, Writing – review & editing.

## Footnotes

^1^In this paper, we use the term “Latina/o/x” to be more inclusive, even though the federal government often uses the term Hispanic. The term “Latina/o/x” is used to refer to individuals with Mexican, Puerto Rican, Cuban, Central or South American, or other Spanish/Portuguese culture or origin, regardless of race, gender, or sexual orientation. We acknowledge that the identity of people with American Indigenous and European ancestry is a complicated subject, and our use of the term “Latina/o/x” also falls short of a proper identification. We also acknowledge the many self-enumeration issues that arise with this specific ethnicity in the United States.
